# The complete mitogenome of *Theloderma albopunctatum* (Liu & Hu 1962) (Anura: Rhacophoridae) from the Karst areas of southwestern China

**DOI:** 10.1080/23802359.2025.2498743

**Published:** 2025-05-16

**Authors:** Jiang Yang, Ming-Le Mao, Cui-Pao He, Qiu-Yuan Lu, Chen-Rui Yan, Ning Xiao, Jiang Zhou

**Affiliations:** a The Administration Bureau of Guangxi, Bangliang Gibbon National Nature Reserve, Jinxi, China; b School of Karst Science, Guizhou Normal University, Guiyang, China; c GuangXi BaiSe Fuluhe National Wetland Park, Baise, China; d School of Life Sciences, Guizhou Normal University, Guiyang, China; e Guiyang Healthcare Vocational University, Guiyang, China

**Keywords:** Mitogenome, *Theloderma albopunctatum*, phylogenetic analysis

## Abstract

This study reports and characterizes the complete mitogenome of *Theloderma albopunctatum*. The mitogenome was 15,780 bp in length and contained 13 protein-coding genes, 22 transfer RNA genes, and two ribosomal RNA genes, with 29.35% A, 25.17% T, 14.24% G, and 31.24% C. The original data were assembled and annotated, and the complete mitochondrial genome was mapped. The phylogenetic tree obtained in this study supports the division of the Rhacophorinae and Buergeriinae subfamilies. This mitogenome provides a valuable resource for understanding evolutionary relationships within the Rhacophoridae family.

## Introduction

**Figure 1.**
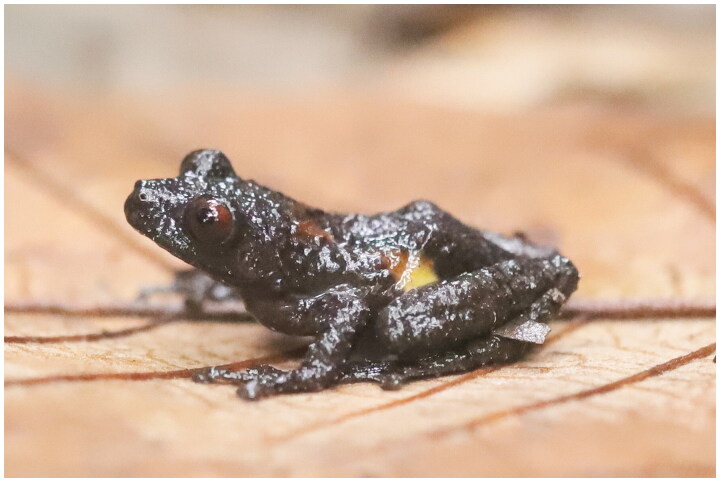
Photo of *T. albopunctatum* by tao luo.

The family Rhacophoridae is highly diverse and widely distributed across Asia (AmphibiaWeb 2024; Frost 2024). Species in this family exhibit a wide range of trait differences and have received considerable attention in the field of biological evolution (Chen et al. 2020). The mossy frogs of the genus *Theloderma* comprise 29 species distributed in southern China, central and northern Vietnam, Laos, and southeastern Cambodia (Frost 2024). *Theloderma albopunctatum* has a body length of approximately 33 mm (Figure 1), and is distributed in the karst mountainous areas of China, including Guangxi, Yunnan, and Hainan (AmphibiaChina 2024). Previous phylogenetic studies have supported *Theloderma* as a sister clade of Nyctixalus (Poyarkov et al. 2018), *Theloderma* can be further divided into two subgenera (*Stelladerma* and *Theloderma*) (Poyarkov et al. 2015) and seven species (Luo et al. 2023). This wide distribution suggests the existence of cryptic species (Luo et al. 2023), though genetic markers remain insufficient for identifying geographic populations. Currently, there are no complete mitochondrial genome reports of the genus *Theloderma*. To fill this gap in knowledge, we sequenced the complete mitogenome of *T. albopunctatum*. This is the first report of the complete mitogenome of the genus *Theloderma* mitogene and will contribute to understanding phylogenetic relationships between *Theloderma* and other genera in the family Rhacophoridae.

## Materials and methods

### Sample collection

In June 2021, approval was obtained from the Bangliang Gibbon National Nature Reserve to conduct biodiversity surveys. Specimen No. 1 was collected from the Bangliang Gibbon National Nature Reserve, Jingxi City, Guangxi Province, China (22.91906924°N, 106.50275230°E; elevation, 827 m). After morphological identification, muscle samples were extracted from the posterior thigh limb of the specimen for genomic analysis and preserved at −20 °C in 95% alcohol at Guizhou Normal University, Guiyang City, Guizhou Province, China (sample ID: GZNU20210715002, http://gznu.edu.cn; contact person: Huai-Qing Deng; e-mail: deng2004616@163.com).

## Methods

Genomic DNA was extracted from 95% ethanol-preserved tissue using the cetyltrimethylammonium bromide method (Allen et al. 2006). The mitogenome was sequenced by Tsingke Biotechnology Co., Ltd. (Chengdu, China) using an Illumina Novaseq 6000 platform (Illumina, San Diego, CA, USA) with 150 bp paired-end reads. Sequencing generated 5.7 G of raw data, which were filtered using SOAPnuke 1.3 (Chen et al. 2018) to obtain 5.4 G of clean data. Clean data were assembled de novo using the Mitoz v. 2.3 software (Supplementary Figure S1). The splicing results were compared with the close reference genome using BLASTN (version: BLAST 2.2.30+; parameter: -value 1e-5) to determine the candidate sequence assembly results. The assembled mitogenome was annotated with genes using MITOS2 (Bernt et al. 2013) and uploaded to the National Center for Biotechnology Information (NCBI) under the accession number OR726341. A circular genome map was generated using Proksee (https://proksee.ca/; Grant et al. 2023). We downloaded 22 mitogenomes from NCBI for molecular analysis, including 11 species of Rhacophorinae and two outgroups. The nucleotide sequences of 13 protein-coding genes (PCGs), 22 tRNAs, and 2 rRNAs from these species were aligned using MAFFT 7.471 (Katoh and Standley 2013) in PhyloSuite 1.2.2 (Zhang et al. 2020) to select the best-fit model (GTR + I + G). Phylogenetic trees were reconstructed based on best-fit partitioning and nucleotide substitution models using the maximum likelihood method in IQ-tree 2.0.4, running 2000 ultrafast bootstrap replicates.

## Results

**Figure 2.**
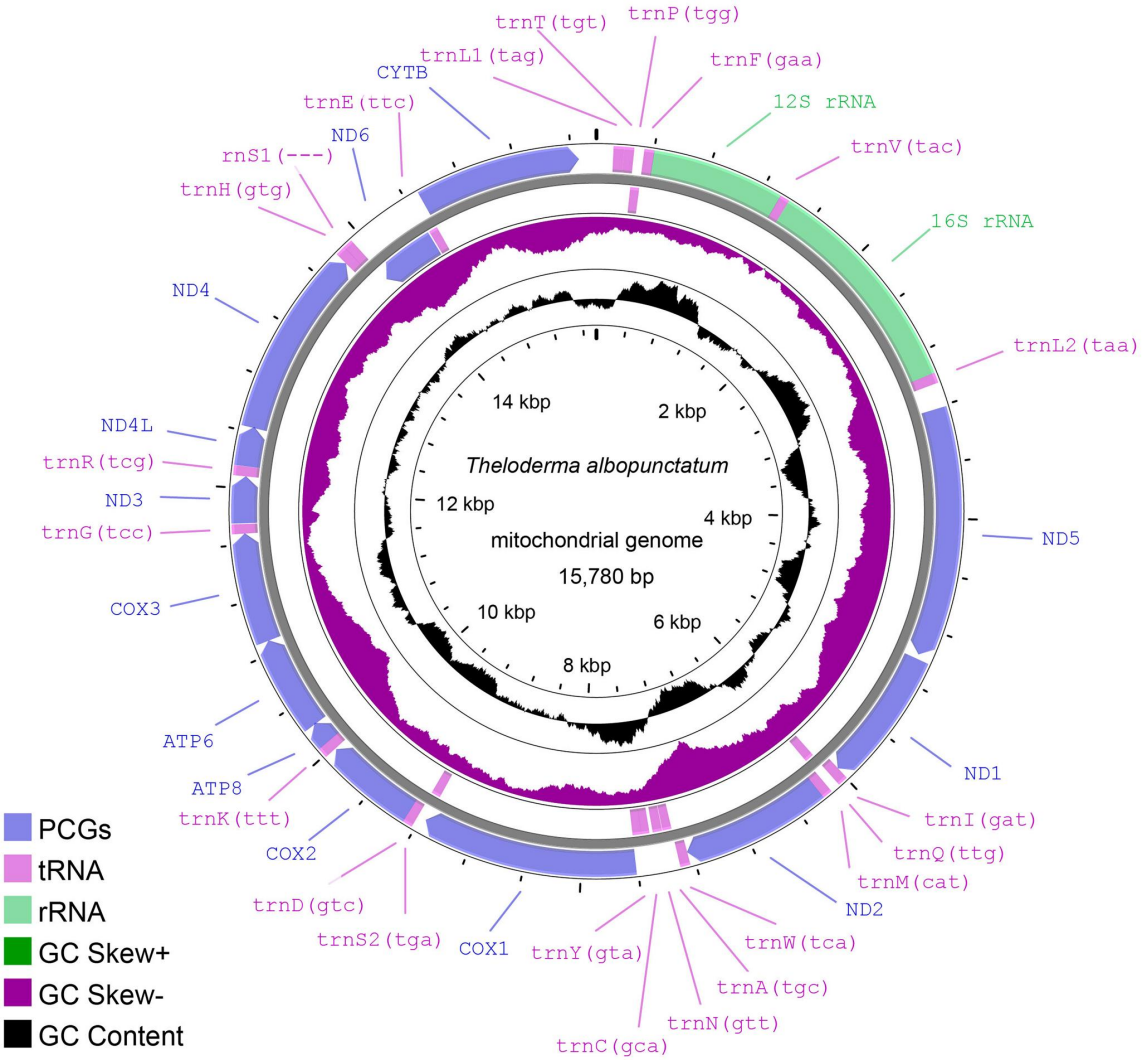
The genome map of the *T. albopunctatum* mitogenome with 13 PCGs, 22 tRNAs, and 2 rRNAs. The inner circle represents the average GC content, while the outer ring shows the reverse and forward strands. Proksee (Grant et al. 2023) was used to create the drawing.

The complete mitogenome of *T. albopunctatum* was 15,780 bp in length (Figure 2) and contained 13 PCGs, 22 tRNA genes, and two rRNA genes, with 29.35% A, 25.17% T, 14.24% G, and 31.24% C (54.52% A + T and 45.48% C + G). The following genes are encoded on the L-strand: *nad6*, *trnQ*(ttg), *trnA*(tgc), *trnN*(gtt), OL, *trnC*(gca), *trnY*(gta), *trnS*(tga), *trnE*(ttc), and *trnP*(tgg); the remaining genes are encoded on the H-strand, which is similar to other typical amphibian mitogenomes (Huang et al. 2019b). Among the PCGs, the largest gene was *nad5*, and the smallest was *atp8*. Among the 13 PCGs, *cox1*, *nad1*, *nad2*, and *nad3* used GTG, CTT, ATA, and ATT as the start codons, respectively, and the remaining nine PCGs used ATG as the start codon. The genes *nad4L*, *nad6*, and *cytb* have TAA as the stop codon; *cox2*, *nad5*, *nad2*, and *atp8* have TAG as stop codons; *atp6*, *cox3*, *nad1*, *nad3*, and *nad4* have T as an incomplete stop codon; and *cox1* uses AGG as the stop codon. The lengths of the 16S and 12S rRNA sequences were 1572 and 928 bp, respectively. The lengths of the 22 tRNA genes ranged between 64 and 73 bp.

## Discussion and conclusion

The results of this study show that the mitogenome sequences of *T. albopunctatum* are similar to those of other species in the subfamily Rhacophorinae in terms of gene arrangement and nucleotide composition. The mitogenome of *T. albopunctatum* was slightly shorter than that of the reference species of the same subfamily, whereas the lengths of the 16S and 12S rRNA genes were similar, which is consistent with the results of Nguyen et al. (2015).

**Figure 3.**
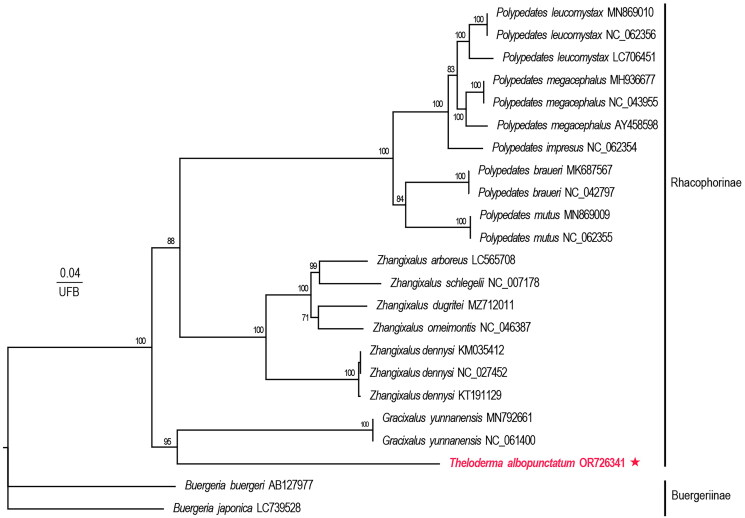
Phylogenetic tree based on the reconstructed mitogenome. Numbers to the left of each node indicate ultrafast bootstrap values (UBP) for maximum likelihood analysis. The specimens of this study are marked in red with a five-pointed star in the figure. The following sequences were used: *Polypedates leucomystax* MN869010, *P. leucomystax* NC062356, *P. leucomystax* LC706451, *P. megacephalus* MH936677 (Huang et al. 2019a), *P. megacephalus* NC043955, *P. megacephalus* AY458598 (Zhang et al. 2005), *P. impresus* NC062354, *P. braueri* MK687567 (Huang et al. 2019b), *P. braueri* NC042797, *P. mutus* MN869009, *P. mutus* NC062355, *zhangixalus arboreus* LC565708 (Inagaki et al. 2020), *Z. schlegelii* NC007178 (Sano et al. 2005), *Z. dugritei* MZ712011, *Z. omeimontis* NC046387 (Fu et al. 2019), *Z. dennysi* KM035412 (Li et al. 2021), *Z. dennysi* NC027452 (Li et al. 2021), *Z. dennysi* KT191129 (Huang et al. 2016), *gracixalus yunnanensis* MN792661, *G. yunnanensis* NC061400, *theloderma albopunctatum* OR726341 (this study, in red), *buergeria buergeri* AB127977 (Sano et al. 2004), *B. japonica* LC739528 (Asaeda et al. 2023).

The phylogenetic tree obtained in this study supports the division of the subfamilies Rhacophorinae and Buergeriinae (ultrafast bootstrap (UFB) = 100%; Figure 3). Furthermore, the phylogenetic analysis confirmed that *Theloderma* was closely related to *Gracixalus* (UFB = 95%; Figure 3), which agrees with the results of Chen et al. (2020). To the best of our knowledge, this is the first report of the sequence, assembly, and annotation of the complete mitogenome of *T. albopunctatum*, providing an opportunity to explore the phylogenetic relationships between *T. albopunctatum* and other species in the subfamily Rhacophorinae. Furthermore, this study provides a reference and basis for systematic classification of the genus *Theloderma*. However, the molecular evidence inferred in this study is limited, and more mitogenomic information on other *Theloderma* species is necessary to elucidate the evolutionary relationships within the major lineages of Rhacophoridae.

## Supplementary Material

Supplemental Material

Supplemental Material

## Data Availability

The genome sequence data that support the findings of this study are openly available in GenBank of NCBI at (https://www.ncbi.nlm.nih.gov/) under the accession on. OR726341. The associated BioProject, SRA, and Bio-sample numbers are PRJNA1031391, SRR26493694, and SAMN37935459, respectively.

## References

[CIT0001] Allen GC, Flores-Vergara MA, Krasynanski S, Kumar S, Thompson WF. 2006. A modified protocol for rapid DNA isolation from plant tissues using cetyltrimethylammonium bromide. Nat Protoc. 1(5):2320–2325. doi:10.1038/nprot.2006.384. 17406474

[CIT0002] AmphibiaChina. 2024. The database of Chinese amphibians. Kunming: Kunming Institute of Zoology (CAS). [accessed 2024 Nov 23]. Electronic Database accessible at http://www.amphibiachina.org/.

[CIT0003] AmphibiaWeb. 2024. Information on amphibian biology and conservation. Berkeley (CA). [accessed 2024 Nov 23]. Available from: https://www.amphibiachina.org/.

[CIT0004] Asaeda Y, Shiraga K, Suzuki M, Sambongi Y, Ogino H, Igawa T. 2023. Rapid and collective determination of the complete "hot-spring frog" mitochondrial genome containing long repeat regions using Nanopore sequencing. PLoS One. 18(10):e0280090. doi:10.1371/journal.pone.0280090. 37906558 PMC10617713

[CIT0005] Bernt M, Donath A, Jühling F, Externbrink F, Florentz C, Fritzsch G, Pütz J, Middendorf M, Stadler PF. 2013. MITOS: improved de novo metazoan mitochondrial genome annotation. Mol Phylogenet Evol. 69(2):313–319. doi:10.1016/j.ympev.2012.08.023. 22982435

[CIT0006] Chen JM, Prendini E, Wu YH, Zhang BL, Suwannapoom C, Chen HM, Jin JQ, Lemmon EM, Lemmon AR, Stuart BL, et al. 2020. An integrative phylogenomic approach illuminates the evolutionary history of Old World tree frogs (Anura: Rhacophoridae). Mol Phylogenet Evol. 145:106724. doi:10.1016/j.ympev.2019.106724. 31881327

[CIT0007] Chen Y, Chen Y, Shi C, Huang Z, Zhang Y, Li S, Li Y, Ye J, Yu C, Li Z, et al. 2018. SOAPnuke: a MapReduce acceleration-supported software for integrated quality control and preprocessing of high-throughput sequencing data. Gigascience. 7(1):1–6. doi:10.1093/gigascience/gix120. PMC5788068 29220494

[CIT0008] Frost DR. 2024. Amphibian species of the world: an online reference. Version 6.2. [accessed 2024 Mar 19]. Available from: http://research.amnh.org/vz/herpetology/amphibia.

[CIT0009] Fu C, Wang Q, Hu T, Lei Z, Fan H, Zhao T, Zong H. 2019. The complete mitochondrial genome of Omei Treefrog (*Rhacophorus omeimontis*). Mitochondrial DNA B Resour. 5(1):300–301. doi:10.1080/23802359.2019.1698334. 33366529 PMC7720942

[CIT0010] Grant JR, Enns E, Marinier E, Mandal A, Herman EK, Chen C, Graham M, Van Domselaar G, Stothard P. 2023. Proksee: in-depth characterization and visualization of bacterial genomes. Nucleic Acids Res. 51(W1):W484–W492. doi:10.1093/nar/gkad326. 37140037 PMC10320063

[CIT0011] Huang A, Li HJ, Luo HD, Ni QY, Yao YF, Xu HL, Zeng B, Li Y, Wei ZM, Zhang MW. 2019b. The complete mitochondrial genome of the tree frog, *Polypedates braueri* (Anura, Rhacophoridae). Mitochondrial DNA Part B. 4(1):1739–1740. doi:10.1080/23802359.2019.1607594.

[CIT0012] Huang A, Liu S, Li HJ, Luo HD, Ni QY, Yao YF, Xu HL, Zeng B, Li Y, Wei ZM, et al. 2019a. The revised complete mitogenome sequence of the tree frog *Polypedates megacephalus* (Anura, Rhacophoridae) by next-generation sequencing and phylogenetic analysis. PeerJ. 7(2):e7415. doi:10.7717/peerj.7415. 31396450 PMC6679912

[CIT0013] Huang M, Lv T, Duan R, Zhang S, Li H. 2016. The complete mitochondrial genome of *Rhacophorus dennysi* (Anura: Rhacophoridae) and phylogenetic analysis. Mitochondrial DNA A DNA Mapp Seq Anal. 27(5):3719–3720. doi:10.3109/19401736.2015.1079873. 26329505

[CIT0014] Inagaki H, Haramoto Y, Kubota HY, Shigeri Y. 2020. Complete mitochondrial genome sequence of Japanese forest green tree frog (*Rhacophorus arboreus*). Mitochondrial DNA B Resour. 5(3):3347–3348. doi:10.1080/23802359.2020.1820396. 33458164 PMC7782537

[CIT0015] Katoh K, Standley DM. 2013. MAFFT multiple sequence alignment software version 7: improvements in performance and usability. Mol Biol Evol. 30(4):772–780. doi:10.1093/molbev/mst010. 23329690 PMC3603318

[CIT0016] Li Y, Zhang H, Wu X, Li D, Yan P, Wu X. 2021. The complete Mitochondrial genome of *Rhacophorus dennysi* (Anura: rhacophoridae) with novel gene arrangements and its phylogenetic implications. PJZ. 53(6):2013–2019. doi:10.17582/journal.pjz/20190901010935.

[CIT0017] Luo T, Zhao XR, Lan CT, Li W, Deng HQ, Xiao N, Zhou J. 2023. Integrated phylogenetic analyses reveal the evolutionary, biogeographic, and diversification history of Asian warty treefrog genus *Theloderma* (Anura, Rhacophoridae). Ecol Evol. 13(12):e10829. doi:10.1002/ece3.10829. 38145017 PMC10739124

[CIT0018] Nguyen TT, Matsui M, Eto K. 2015. Mitochondrial phylogeny of an Asian tree frog genus *Theloderma* (Anura: rhacophoridae). Mol Phylogenet Evol. 85:59–67. doi:10.1016/j.ympev.2015.02.003. 25683047

[CIT0019] Poyarkov NA, Jr, Kropachev II, Gogoleva SS, Orlov NL. 2018. A new species of the genus *Theloderma Tschudi*, 1838 (Amphibia: anura: rhacophoridae) from Tay Nguyen Plateau, central Vietnam. Zoological Research. 39(3):158–184. 29683110 10.24272/j.issn.2095-8137.2018.018 PMC5968860

[CIT0020] Poyarkov NA, Jr, Orlov NL, Moiseeva AV, Pawangkhanant P, Ruangsuwan T, Vassilieva AB, Gogoleva SS. 2015. Sorting out moss frogs: mtDNA data on taxonomic diversity and phylogenetic relationships of the Indochinese species of the genus *Theloderma* (Anura, Rhacophoridae). Russ J Herpetol. 22(4):241–280.

[CIT0021] Sano N, Kurabayashi A, Fujii T, Yonekawa H, Sumida M. 2004. Complete nucleotide sequence and gene rearrangement of the mitochondrial genome of the bell-ring frog, *Buergeria buergeri* (family Rhacophoridae). Genes Genet Syst. 79(3):151–163. doi:10.1266/ggs.79.151. 15329496

[CIT0022] Sano N, Kurabayashi A, Fujii T, Yonekawa H, Sumida M. 2005. Complete nucleotide sequence of the mitochondrial genome of Schlegel’s tree frog *Rhacophorus schlegelii* (family Rhacophoridae): duplicated control regions and gene rearrangements. Genes Genet Syst. 80(3):213–224. doi:10.1266/ggs.80.213. 16172533

[CIT0023] Zhang D, Gao F, Jakovlić I, Zou H, Zhang J, Li WX, Wang GT. 2020. PhyloSuite: an integrated and scalable desktop platform for streamlined molecular sequence data management and evolutionary phylogenetics studies. Mol Ecol Resour. 20(1):348–355. doi:10.1111/1755-0998.13096. 31599058

[CIT0024] Zhang P, Zhou H, Chen YQ, Liu YF, Qu LH. 2005. Mitogenomic perspectives on the origin and phylogeny of living amphibians. Syst Biol. 54(3):391–400. doi:10.1080/10635150590945278. 16012106

