# Supplementary figures and images for: The complete mitogenome of Theloderma albopunctatum (Liu & Hu 1962) (Anura: Rhacophoridae) from the Karst areas of southwestern China
Source: Mitochondrial DNA B Resour. 2025 May 16;10(6):499–503. doi: 10.1080/23802359.2025.2498743 (PMC12086936; doi:10.1080/23802359.2025.2498743)

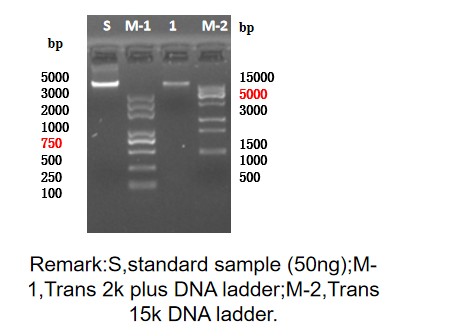

Supplement: Supplemental Material [file TMDN_A_2498743_SM8863.jpg]

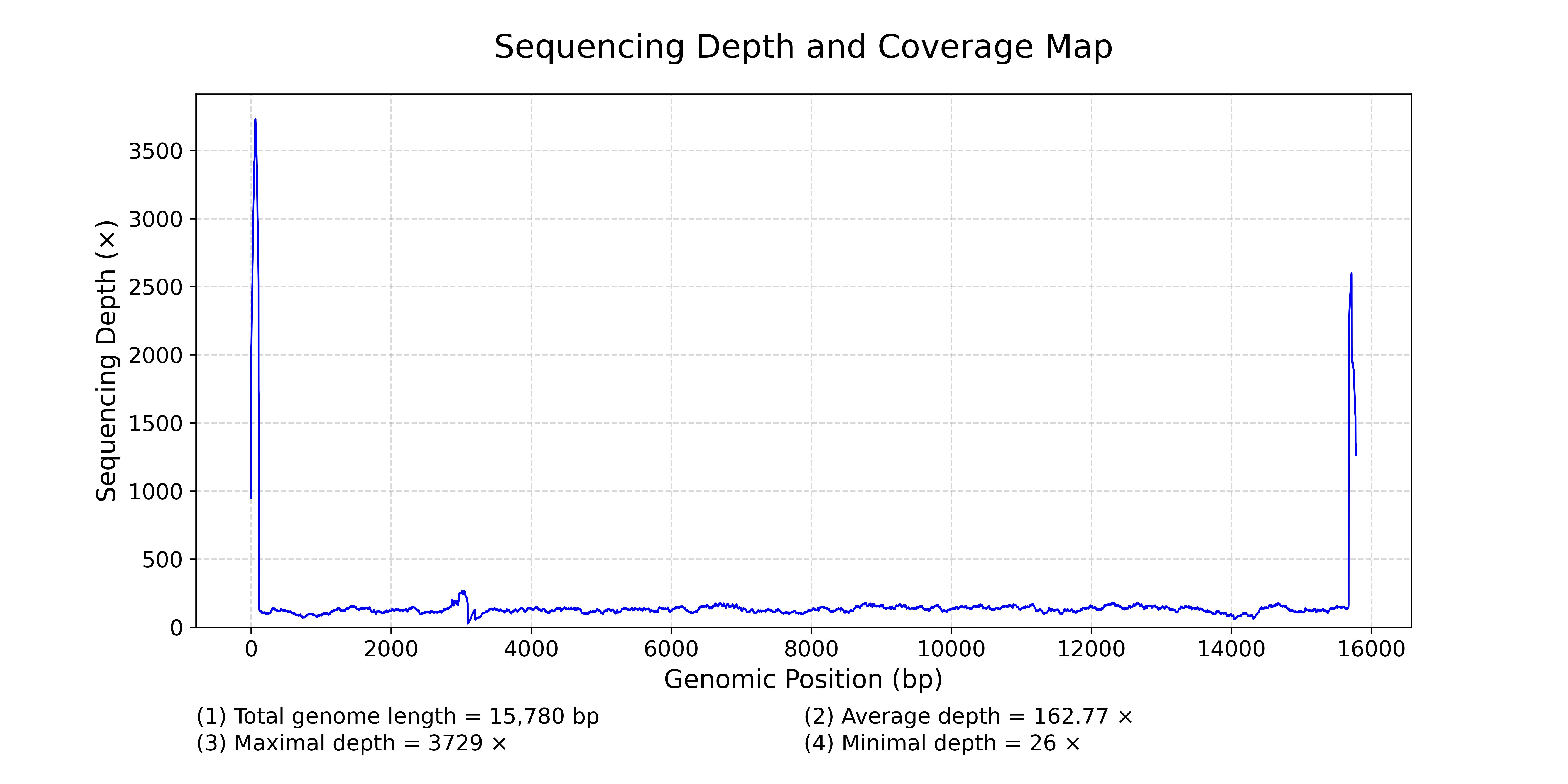

Supplement: Supplemental Material [file TMDN_A_2498743_SM8861.jpg]
